# Risk and Protective Factors of Food Insecurity Among Children in Low-Income Families in the United States: Application of the Multiple Disadvantage Model

**DOI:** 10.3390/ejihpe16100156

**Published:** 2026-10-01

**Authors:** Tyrone C. Cheng, Celia C. Lo

**Affiliations:** 1 School of Social Work, University of Alabama, Little Hall, Tuscaloosa, AL 35401, USA; 2 Peraton, Seaside, CA 93955, USA; celiaclo@yahoo.com

**Keywords:** food insecurity, low-income families, health, mental health, neighborhood, racial discrimination

## Abstract

This study investigated relationships between low-income children’s food insecurity and five factors: social disorganization, social structural factors, social relationships, health/mental health, and access to services. A sample of 12,215 low-income children and parents was extracted from the 2021 National Survey of Children’s Health. Logistic regression results showed the likelihood of such children’s food insecurity to be associated positively with several factors: racial discrimination, child aged 6–10 years, child aged 11–13 years, child aged 14–17 years, Black child, child’s chronic health condition, family member mental health problem, Supplemental Nutrition Assistance Program participation, receipt of Special Supplemental Nutrition Program for Women, Infants, and Children, and lunch program participation. As well, children’s food insecurity likelihood was associated negatively with parents’ age and education, neighbor support, parents’ general health and mental health, and receipt of Temporary Assistance for Needy Families. Research implications include interventions at the individual level (e.g., screening for food insecurity, treatment of sick or mentally ill family members), community level (e.g., food pantries, neighbor support network), and macro level (e.g., advocating for racial/ethnic minorities, expanding cash and food benefits).

## 1. Introduction

Food insecurity of a household is defined as limited ability to acquire adequate food through a lack of money or other resources, reducing food intake and disrupting eating patterns (Coleman-Jensen et al., 2022; United States Department of Agriculture, 2024). Studies with low-income families have found that 6.6% to 15.0% of families had suffered food insecurity (Daundasekara et al., 2024; Driver et al., 2023; Oh, 2023). It is important to examine any factors as well beyond economic status that may relate to food insecurity among low-income families with children.

### Literature Review

**Figure 1:**
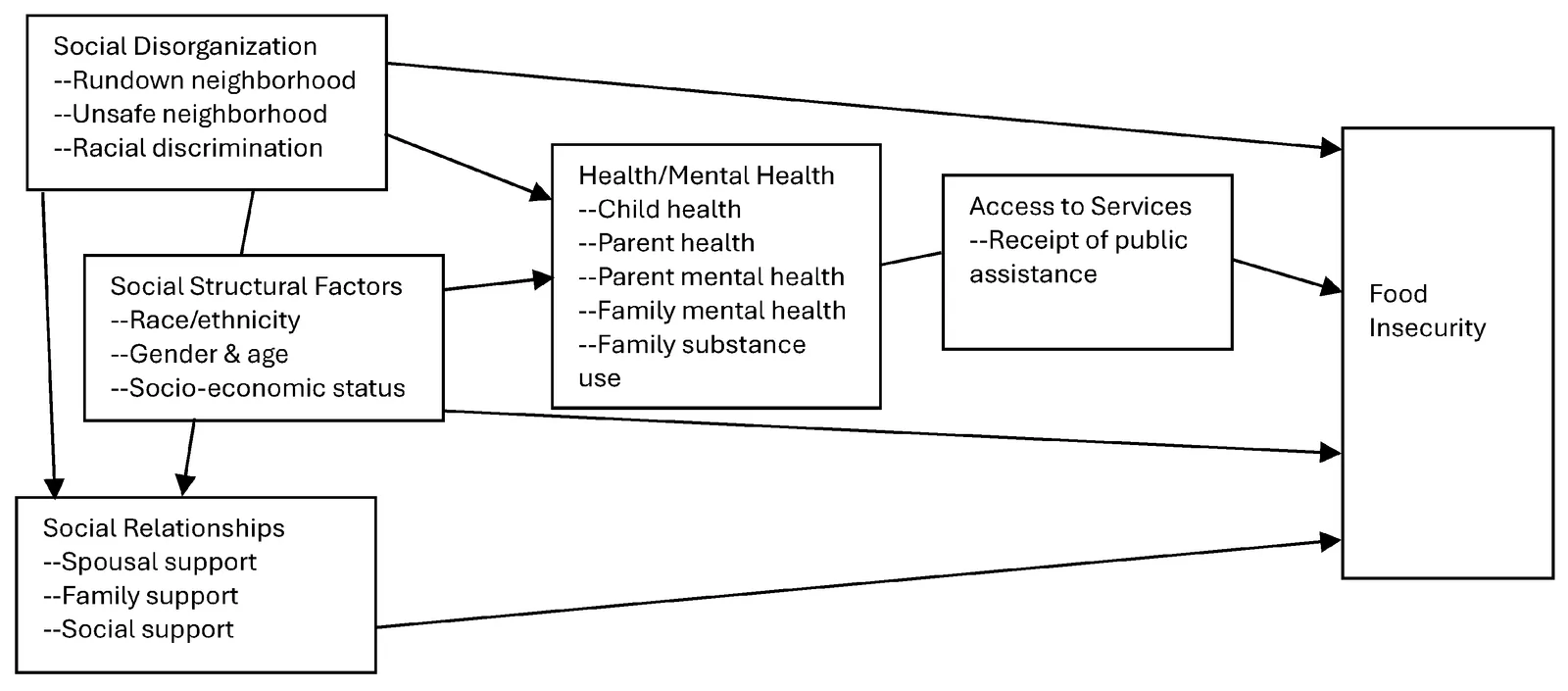
The multiple disadvantage model explaining low-income children’s food insecurity.

The present study applied the multiple disadvantage model to investigate factors in low-income families’ experience of food insecurity (see Figure 1). The multiple disadvantage model suggests that socioeconomic disadvantages (such as social disorganization and certain social structural factors) and distress associated with them undesirably affect individuals’ social relationships with family, peers, and others, as well as their health and mental health; furthermore, they relate to their access to services. The multiple disadvantage model has been applied to explain low-income children’s health (Cheng & Lo, 2024) and participation in school lunch programs (Cheng & Lo, 2026). The present research used the model to examine whether five socioeconomic disadvantage factors (social disorganization, social structural factors, social relationships, health/mental health, and access to services) accounted for low-income families’ food insecurity.

Social disorganization, such as living in unkempt, crime-ridden, unsafe neighborhoods, has been associated with food insecurity of low-income families (Daundasekara et al., 2024; Miller et al., 2021). As well, structural and historical racism have persistently upset individuals of minority race/ethnicity (Lo et al., 2013). Racial discrimination distress’s social structure and generates residential instability, leaving communities socially disorganized (Lo et al., 2013). Racial/ethnic minority families who experience racial discrimination and structural racism can themselves be distressed and exhibit food insecurity (Anglin, 2023; Bowen et al., 2021; Chaparro et al., 2022; Ngo et al., 2024; Odoms-Young & Bruce, 2018; Phojanakong et al., 2019; Yang et al., 2023).

Some social structural factors—child race/ethnicity, age, and gender as well as parent age, education, employment, and family income—appear related to food insecurity of low-income families. While one study of Supplemental Nutrition Assistance Program (SNAP) recipients found Asians to be less likely than Whites to be food-insecure (Samuel et al., 2023), another study reported no association between race/ethnicity and food insecurity of low-income families (Driver et al., 2023). Some studies with the general population demonstrated that food insecurity had no association with child gender or age (Turner et al., 2022). Another study, however, did observe a negative association between child age and food insecurity (Johnson & Markowitz, 2018). Furthermore, a different study of SNAP recipients found older adults less likely to experience food insecurity than younger adults (Samuel et al., 2023). As would be expected, studies have found negative associations between family income and food insecurity (Coughenour et al., 2024; Crossa et al., 2024; Livings et al., 2023; Mazzeo et al., 2023; Mortensen & Jeon, 2023; Vericker et al., 2023). One study with low-income families did not find an association between parent education level and food insecurity (Driver et al., 2023). In addition, research with SNAP users (Mabli et al., 2023) showed that parents’ unemployment (a disadvantage) increases a family’s likelihood of food insecurity.

Research suggests that supportive social relationships can lessen distress from socioeconomic disadvantages, although one study with low-income families found no association between single parenthood and food insecurity, despite the implied absence of spousal support (Driver et al., 2023). Indeed, research has shown that conflictual or violent spousal relationships raise the risk of food insecurity (Johnson & Markowitz, 2018; Ngo et al., 2024). In turn, when relatives, neighbors, and/or friends provide social support to low-income families, participation in public food assistance programs is reduced (Cheng & Tang, 2016). Such support also cuts the likelihood of food insecurity in low-income families (Driver et al., 2023).

Children’s physical health problems as well as parents’ physical and mental health problems can contribute to food insecurity. Prior studies with low-income families have reported a negative association between children’s and parents’ physical health and the risk of food insecurity (Driver et al., 2023; Samuel et al., 2023). Moreover, it is reported that just a tenth of parents with depression pursue professional help for it (Cheng & Lo, 2016; Woolhouse et al., 2009). Among the other 90 percent, at least some self-medicate by misusing substances, which worsens their emotional condition and their ability to manage a household and parent children. A study with the general population showed alcohol-use disorder to raise adults’ risk of food insecurity (Nagata et al., 2021).

Limited access to services or resources can disadvantage low-income families and increase risk of food insecurity among them (Park et al., 2024). Studies have found family enrollment in SNAP to be associated in a positive direction with food insecurity, which implies that food-insecure low-income families do access needed services (Coughenour et al., 2024; Driver et al., 2023; Mabli et al., 2023). On the other hand, several studies have reported that school lunch/meal program participation is limited by parents’ or children’s sense of shame; pick-up difficulties; and lack of knowledge of program availability (Chaparro et al., 2023; Gagliano et al., 2023; Vericker et al., 2023).

The reviewed literature comprised just a small number of studies of low-income families’ food insecurity. Allowing the multiple disadvantage model to offer a different view of low-income children’s food insecurity, the present study hypothesized that (1) low-income families’ food insecurity would be associated positively with social disorganization (i.e., rundown, unsafe neighborhoods; racial discrimination), with social structural factors (i.e., racial/ethnic minority status), with social relationships (i.e., single motherhood), parent mental health problem or substance use; and that (2) low-income families’ food insecurity would be associated negatively with social structural factors (i.e., parent education, parent employment, and family income), with social relationships (i.e., spousal support, family support, and social support), with child and parent physical health, and with access to services.

## 2. Material and Methods

### 2.1. Sample

This study analyzed secondary data from a nationally representative sample of 12,215 children in low-income families, extracted from the 2021 National Survey of Children’s Health (NSCH) dataset. NSCH participants included 50,892 children and their caregivers in the United States. Through mailed and emailed questionnaires and phone interviews, the original NSCH researchers collected information from caregivers on food security, family and social relationships, physical health and mental health, access to services, and neighborhood maintenance and security (United States Bureau of Census, 2022). The present sample included only those child participants and parents (i.e., biological parents or stepparents) whose family income reportedly was below 200% of the federal poverty level. A university institutional review board exempted the present secondary analysis from the approval process. The authors report there are no competing interests to declare.

### 2.2. Measures

The outcome variable, food insecurity (yes/no), indicated whether a family had reportedly sometimes or often been unable to afford enough to eat in the preceding 12 months. Five groups of explanatory variables were employed: social disorganization variables, social structural factors, social relationship variables, health/mental health variables, and access-to-services factors.

The first group of explanatory variables comprised social disorganization factors. *Rundown neighborhood* (yes/no) indicated whether a family’s neighborhood was described as “poorly kept or rundown housing,” “litter or garbage on street or sidewalk,” or “vandalism such as broken windows or graffiti.” *Safe neighborhood* assessed parents’ level of agreement with neighborhoods’ safety for their children, using a 4-point scale: 1 (*definitely disagree*), 2 (*somewhat disagree*), 3 (*somewhat agree*), and 4 (*definitely agree*). *Racial discrimination* (yes/no) denoted whether a child had ever been treated or judged unfairly because of her/his racial/ethnic background.

The second group of explanatory variables characterized children’s and parents’ social structural factors. The dummy variables Hispanic, Black, Asian, other racial/ethnic minority, and non-Hispanic White (the reference) described child race/ethnicity. Other demographic variables were girl (versus boy), child aged 5 years or less (the reference), child aged 6–10 years, child aged 11–13 years, child aged 14–17 years, and parent age (ranging 18–75 years). Parent education was measured using nine levels: 1 (8th grade or below), 2 (9th–12th grade), 3 (graduated high school or GED), 4 (vocational school), 5 (some college), 6 (associate degree), 7 (undergraduate degree), 8 (master’s degree), and 9 (doctoral or professional degree). Employed parent (yes/no) indicated if a parent had been employed in 50 of the 52 weeks preceding the NSCH interview. The ratio of each family’s income to the federal poverty level (FPL) was represented by two dummy variables: family income at or below 100% of FPL and family income at 101–200% of FPL (the reference).

The third group of explanatory variables represented social relationships. Single mother (yes/no) denoted households headed by an unmarried female parent. Family cohesiveness was the total score of three survey items regarding how family members handled their problems—“[we] talk together about what to do,” “[we] work together to solve our problems,” and “[we] have strengths to draw on.” Using a four-point scale comprising 1 (none of the time), 2 (some of the time), 3 (most of the time), and 4 (all the time), the three items yielded a Cronbach’s alpha of 0.91; a higher total score indicated stronger family cohesiveness. Family support (yes/no) indicated whether a parent received emotional support from a spouse/partner, other family members, and/or friends. Peer/religious group support (yes/no) denoted whether a parent received emotional support from a support group of peers or from a religious group. Neighbor support was the total score of three survey items measuring a parent’s perception of other adults in the neighborhood: (a) neighbors provide help to others, (b) neighbors watch out for each other’s children, and (c) neighbors know where to get help. Using a four-point scale comprising 1 (definitely disagree), 2 (somewhat disagree), 3 (somewhat agree), and 4 (definitely agree), the Cronbach’s alpha for the three items was 0.79; a higher total score suggested a stronger network of supportive neighbors.

Five explanatory variables represented individuals’ health and mental health. Child chronic health condition(s) (yes/no) described whether, in the 12 months preceding an NSCH interview, a child had exhibited a chronic problem/difficulty with breathing, using their hands, coordination or moving around, physical pain, swallowing, or digestion. Parent general health was measured with five responses: 5 (excellent), 4 (very good), 3 (good), 2 (fair), and 1 (poor). The same five-point scale measured parent mental health. Family mental health problem (yes/no) denoted whether a child had ever lived with someone who was mentally ill. Family substance-use problem (yes/no) indicated whether a child had ever lived with someone who had a problem with alcohol and/or drug use.

Child’s access to services was measured using family participation in public assistance programs, via four variables: receipt of TANF (yes/no), receipt of SNAP (yes/no), receipt of WIC (yes/no), and receipt of Lunch Program (yes/no). These variables described, respectively, family participation in the federal benefits programs Temporary Assistance for Needy Families (TANF), Supplemental Nutrition Assistance Program (SNAP), Special Supplemental Nutrition Program for Women, Infants, and Children (WIC), and free or reduced-cost school-based breakfast and/or lunch, in the 12 months preceding the NSCH interview.

### 2.3. Data Analysis

With food insecurity as the binary outcome variable, the present study used STATA logistic regression to estimate linearized variance with robust standard errors. During analysis, the sampling weights specified in the NSCH data set were also employed. Where data on continuous measures were missing, the present researchers substituted the sample-wide average for the relevant variable in calculations. Where data quantifying dichotomous (yes/no) measures were missing, the present researchers supplied a “no” response. All explanatory variables were entered simultaneously in the logistic regression modeling. Preliminary analyses of tolerance statistics (0.53 or higher) and correlations (−0.33 ≤ r ≤ 0.61; *p* < 0.01) indicated no multicollinearity problems among the explanatory variables. To further strengthen understanding of the explanatory variables’ relationships with the outcome variables, the present study also explored and reported statistically significant interaction terms between some explanatory variables.

## 3. Results

### 3.1. Descriptive Statistics

Of the 12,215 low-income children, 7.1% experienced food insecurity, 32.1% resided in rundown neighborhoods, and 4.5% had been racially discriminated against (see Table 1). On average, parents reported they “somewhat agreed” (score of 3.5) that their neighborhoods were safe for their children. Of all the low-income children, 52.2% were White, 22.3% were Hispanic, 11.1% were Black, 5.8% were Asian, and 8.6% were other ethnicity/race; moreover, 47.3% were girls, and 38.1% were aged 5 years or below, with 23.9% aged 6–10 and 15.3% aged 11–13 and 22.7% aged 14–17. In the sample, the average parent age was 38.5 years, and the average education level was 5.0 (some college completed). Moreover, 28.5% of parents were single mothers, 62.8% were employed, and 43.7% of parents’ family incomes were at or below 100% of FPL. On average, family cohesiveness measured 10.4 (of 12.0 possible) and neighbor support was 9.4 (of 12.0 possible). Of all the parents, 71.8% received family support and 27.1% received peer/religious group support.

Of the 12,215 low-income children, 21.0% reportedly had chronic health condition(s); parents’ health was good, on average (measuring 3.7), as was their mental health (measuring 3.8). Of the sampled children’s family members, 10.9% reportedly had mental health problems, and 9.7% had substance-use problems within the 12 months specified. Of the interviewed families, 8.3% received TANF benefits, 36.3% received SNAP benefits, and 19.9% received WIC benefits; of the children, 50.4% qualified for free or reduced-cost breakfast and/or lunch at school.

### 3.2. Multivariate Analysis Results

Multivariate analysis results confirmed that the hypothesized model differed significantly from the null model (Wald’s χ^2^ = 500.77, *p* < 0.01; see Table 2). Low-income children’s odds of food insecurity were associated positively with experience of racial discrimination (OR = 2.18; *p* < 0.01), but their odds of being food-insecure showed no association with living in a rundown neighborhood or in a safe neighborhood. Additionally, the children’s odds of food insecurity were associated positively with the variables of child aged 6–10 years (OR = 1.73, *p* < 0.01), child aged 11–13 years (OR = 1.47, *p* < 0.1), child aged 14–17 years (OR = 1.47, *p* < 0.1), and Black child (OR = 1.48, *p* < 0.1). In turn, parents’ food insecurity odds were negatively associated with parent age (OR = 0.98, *p* < 0.1), parent education (OR = 0.90, *p* < 0.01), and neighbor support (OR = 0.88, *p* < 0.01). Other social structural factors and social relationship variables employed in this study showed no significant association with such odds.

Closer examination of the data found an association in the positive direction between the outcome variable and an interaction term between two variables, Black and child aged 5 years or below (OR = 3.53, *p* < 0.01). The data also showed that food insecurity odds were negatively associated with the interaction between neighbor support and child aged 11–13 years (OR = 0.86, *p* < 0.01). An interaction term between the variables neighbor support and racial discrimination (OR = 1.18, *p* < 0.05) in turn showed an association in positive direction with the outcome variable.

The present analysis also observed a negative association between the outcome (odds of food insecurity among low-income children) and parent general health (OR = 0.81, *p* < 0.05) and mental health (OR = 0.76, *p* < 0.01). In contrast, child chronic health condition (OR = 1.50, *p* < 0.01) and family mental health problem (OR = 1.69, *p* < 0.01) were found to be associated positively with their food insecurity. Additionally, participation in TANF (OR = 0.57, *p* < 0.01) was associated negatively with the outcome variable, while having SNAP (OR = 1.54, *p* < 0.05), WIC (OR = 1.42, *p* < 0.1), or school-based subsidized meals (OR = 1.94, *p* < 0.01) was associated positively with the odds of children’s food insecurity. Examining the data even more closely, the present researchers found that an interaction term between the variables of family mental health problem and child aged 6–10 years (OR = 2.39, *p* < 0.1) was associated positively with the outcome, as was an interaction term between family mental health problem and child aged 14–17 years (OR = 2.48, *p* < 0.01). Finally, an interaction term between qualifying for subsidized school-based meals and experiencing racial discrimination (OR = 3.73, *p* < 0.05) was found to be associated positively with children’s odds of food insecurity.

## 4. Discussion

The present NSCH results from 2021 showed that 7.1% of sampled low-income children reported experiencing food insecurity, a percentage similar to the 6.6% reported in a prior analysis of low-income families (Oh, 2023). Via a further, closer examination of the present data, it was indicated that 8.5–11.5% of the low-income children were food-insecure in the year preceding the NSCH interview despite being enrolled in public assistance programs. That percentage was smaller than percentages reported for SNAP recipients sampled for earlier research, which ranged from 26% to 56% (Mabli et al., 2023; Samuel et al., 2023). That is, according to the present findings, some one-tenth of low-income children experience food insecurity, even when their families are assisted financially by public food and/or cash programs.

Results of the present multivariate analysis offer partial support of the hypothesis, that (1) low-income families’ food insecurity would be associated positively with social disorganization (i.e., rundown, unsafe neighborhoods; racial discrimination), with social structural factors (i.e., racial/ethnic minority status), with social relationships (i.e., single motherhood), parent mental health problem or substance use; and that (2) low-income families’ food insecurity would be associated negatively with social structural factors (i.e., parent education, parent employment, and family income), with social relationships (i.e., spousal support, family support, and social support), with child and parent physical health, and with access to services.

The present research showed experiencing racial discrimination to be the strongest predictive factor in the analysis, increasing low-income children’s odds of food insecurity by 118%. One plausible explanation is that many families of minority race/ethnicity may feel distressed or discouraged by unfair treatment while shopping for food (Krieger et al., 2022; Zenk et al., 2014). In contrast, the present results indicate no significant association between food insecurity and families’ residence in unkempt or unsafe neighborhoods. The present findings imply that racial discrimination adversely affects low-income families’ food security no matter the appearance or degree of safety characterizing their neighborhoods.

Among social structural factors in the present research, only parent age, parent education, child age, and Black child were significantly associated with food insecurity. It was found here that Black children were more likely to experience food insecurity than non-Hispanic White children were. Additional analysis of the sample showed the rate of food insecurity by race/ethnicity as follows: Black, 10.8%; Hispanic, 7.3%; Asian, 4.4%; other racial/ethnic minority, 8.0%; and non-Hispanic White, 6.4%. The present study also found that children 6 years old and older were more likely to experience food insecurity than children 5 years old or younger. The present study speculated that parents would have difficulties in providing sufficient food for school-aged children in low-income families.

Similarly, the present researchers examined an interaction term between the variables Black child and child aged 5 years or below and found it to raise odds of food insecurity by 253%. Such a finding indicates remarkably high odds that Black infants and toddlers will experience food insecurity. Furthermore, the present study found that odds of food insecurity were negatively associated with parents’ greater age and education level. The present study speculated that older, well-educated parents likely had more life experience and skills in maintaining food security. In sum, the present findings argue that social structural factors crucially involved in low-income families’ food insecurity are limited to child age, parent age, parent education, and Black child.

The present analysis also found that support from neighbors was the only significant social relationship to show a negative association with low-income families’ food insecurity. Such a finding implies that the members of low-income families may borrow food from neighbors when they cannot buy it themselves (Driver et al., 2023). Furthermore, the interaction term between neighbor support and children aged 11–13 years was associated with lower odds of food insecurity among low-income children. The present researchers speculate that borrowing food from neighbors helps some parents provide sufficient food for their middle school–aged children. Although the interaction term between racial discrimination and neighbor support increased the odds of food insecurity by 18%, such a level of increase in odds was much lower than that (118%) made by the impact of racial discrimination alone. The present researchers speculate that, again, borrowing food from neighbors would be a helpful strategy for food security for low-income families facing the distress of racial discrimination.

The present results showed that low-income children’s chronic health conditions raise their odds of food insecurity by 50%. Some of their chronic health problems may relate directly to malnutrition and hunger. Moreover, it is quite possible that children’s chronic health conditions generate ongoing distress for their parents, hindering the parents’ ability to maintain household food security. In addition, per the present results, parents’ physical health and mental health were associated negatively with low-income children’s odds of being food insecure. That is, parents in good physical and mental health are relatively better able to secure sufficient food for their children. However, in the present study as well, an association in the positive direction was observed between children’s food insecurity and problematic mental health of their family members. Interaction terms were identified between family mental health problems and children aged 6–10 years and between family mental health problems and children aged 14–17 years; each term demonstrated an association in positive direction with children’s odds of food insecurity. The present study speculated that the social environment of residing with family members with mental health problems would distress the parents, leading to budgeting problems and older children’s food insecurity.

The variables of access to services explored in the present research included one: receipt of TANF, which was found to be negatively associated with odds of child food insecurity. This association implies that TANF assistance helps low-income families afford more food. The present research also found a positive association between low-income children’s food insecurity and their families’ participation in SNAP. Such an association suggests that many food-insecure low-income families do pursue SNAP benefits (Coughenour et al., 2024; Driver et al., 2023; Mabli et al., 2023). Moreover, according to the present findings, enrollment in WIC and in school-based meal programs is associated positively with children’s risk of food insecurity. The findings also suggest that families experiencing greater food insecurity are more likely to apply for and qualify for benefits. On the other hand, receipt of lunch programs’ interaction term with racial discrimination significantly further raised the odds of food insecurity among low-income children by 273%. Such findings suggest that shaming and racial discrimination deter low-income children to use benefits of lunch programs, leading to their food insecurity (Gagliano et al., 2023).

## 5. Conclusions

This application of the multiple disadvantage model to a 2021 data set encompassing a nationally representative sample of low-income families around the U.S. identified important risk and protective factors in low-income children’s food insecurity. Its findings may inform interventions that curb food insecurity among those who experience racial discrimination. For instance, via education and outreach, communities and schools could establish anti-racism and anti-shaming policies, especially for participants in school-based meal benefits. Communities and schools may also promote awareness of food insecurity’s and racial discrimination’s harmful impacts on children, perhaps through activities that foster racial harmony and respect for diversity.

Potentially helpful macro-level interventions may include regulating U.S. workers’ pay to help low-income parents put food on the table (Bowen et al., 2021). They may also include expanding income-support benefits like the federal Earned Income Tax Credit (Odoms-Young & Bruce, 2018) or increasing SNAP benefits (Hamel & Harman, 2023). Reforming federal food programs and social programs could also be advocated, with an eye toward eradication of current policies (federal and state) that function punitively and largely affect parents of minority race/ethnicity (Bowen et al., 2021). Finally, advocacy groups could organize food distributions for low-income families in racial/ethnic minorities, including perhaps a simple program providing free breakfast (Bowen et al., 2021), while local governments could open generous food pantries in low-income neighborhoods (Hamel & Harman, 2023).

Interventions at the individual level could also potentially help food-insecure low-income families. For example, medical professionals could screen children from such families without charge; this would be especially impactful for infants and toddlers of minority race/ethnicity. Additionally, licensed social workers could more readily help food-insecure, low-income parents apply for available cash and food programs, and connect them with local food pantries. Social workers could also inform young parents about food security’s importance and, if applicable, refer for treatment family members who are physically or mentally ill. Finally, social workers could work at the group level to organize networks of neighbors willing to share or loan food.

Although the relationship between food insecurity and some variables (e.g., children’s and parents’ health) may involve bidirectional causation, the present study was limited by its cross-sectional design. Future research will aim to investigate causal relationships by using longitudinal data. Another limitation was that the NSCH dataset did not collect information on low-income families’ responses to food insecurity. To inform practical strategies or interventions, future research will collect information from low-income families about their responses to food insecurity. The third limitation of the food insecurity outcome variable was that it was based on a single yes/no indicator of whether the family sometimes or often could not afford enough to eat during the previous 12 months. Such a measure has a great limitation when it is compared with comprehensive food insecurity measures applied in other studies.

## Figures and Tables

**Table 1:** Descriptive statistics of low-income children (n = 12,215).

	Percent	Mean	Range	sd
Outcome variable				
Food insecurity (yes)	7.1%			
(no)	92.9%			
Social disorganization factors				
Rundown neighborhood (yes)	32.1%			
(no)	67.9%			
Safe neighborhood		3.5	1–4	0.7
Racial discrimination (yes)	4.5%			
(no)	95.5%			
Social structural factors				
Non-Hispanic White	52.2%			
Black	11.1%			
Hispanic	22.3%			
Asian	5.8%			
Other racial/ethnic minorities	8.6%			
Girl	47.3%			
Boy	52.7%			
Child aged 5 years or below	38.1%			
Child aged 6–10 years	23.9%			
Child aged 11–13 years	15.3%			
Child aged 14–17 years	22.7%			
Parent age (years)		38.5	18–75	8.7
Parent education		5.0	1–9	2.0
Employed parent (yes)	62.8%			
(no)	37.2%			
Family income at or below 100% of FPL	43.7%			
Family income at 101–200% of FPL	56.3%			
Social relationships				
Single mother (yes)	28.5%			
(no)	71.5%			
Family cohesiveness		10.4	3–12	1.9
Family support	71.8%			
Peer/religious group support	27.1%			
Neighbor support		9.4	3–12	2.2
Health/mental health				
Child chronic health condition(s) (yes)	21.0%			
(no)	79.0%			
Parent general health		3.7	1–5	1.0
Parent mental health		3.8	1–5	1.0
Family mental health problem (yes)	10.9%			
(no)	89.1%			
Family substance use problem (yes)	9.7%			
(no)	90.3%			
Access to services				
Receipt of TANF (yes)	8.3%			
(no)	91.7%			
Receipt of SNAP (yes)	36.3%			
(no)	63.7%			
Receipt of WIC (yes)	19.9%			
(no)	80.1%			
Receipt of Lunch Program (yes)	50.4%			
(no)	49.6%			

Note: sd—standard deviation.

**Table 2:** Logistic regression results on low-income children’s food insecurity (n = 12,215).

Variables	OR	RSE	90% CI
Social disorganization factors			
Rundown neighborhood (no)	0.99	0.14	0.78–1.26
Safe neighborhood	0.97	0.10	0.81–1.15
Racial discrimination (no)	2.18 ***	0.58	1.40–3.39
Social structural factors			
Black (non-Hispanic White)	1.48 *	0.34	1.02–2.16
Hispanic (non-Hispanic White)	0.97	0.18	0.72–1.31
Asian (non-Hispanic White)	0.83	0.27	0.49–1.43
Other racial/ethnic minorities (non-Hispanic White)	0.93	0.20	0.65–1.32
Girl (boy)	1.13	0.15	0.91–1.40
Child aged 6–10 years (aged 5 years or below)	1.73 ***	0.33	1.26–2.37
Child aged 11–13 years (aged 5 years or below)	1.47 *	0.33	1.01–2.12
Child aged 14–17 years (aged 5 years or below)	1.47 *	0.33	1.02–2.11
Parent age	0.98 *	0.01	0.97–0.99
Parent education	0.90 ***	0.03	0.84–0.95
Employed parent (no)	0.79	0.12	0.61–1.02
Family income at or below 100% of FPL (at 101–200% of FPL)	0.91	0.14	0.71–1.16
Social relationships			
Single mother (no)	0.98	0.16	0.75–1.28
Family cohesiveness	1.02	0.03	0.97–1.08
Family support	0.88	0.14	0.68–1.14
Peer/religious group support	0.88	0.15	0.67–1.17
Neighbor support	0.88 ***	0.03	0.83–0.93
Health/mental health			
Child chronic health condition(s) (no)	1.50 ***	0.21	1.19–1.88
Parent general health	0.81 **	0.07	0.70–0.93
Parent mental health	0.76 ***	0.06	0.66–0.87
Family mental health problem (no)	1.69 ***	0.31	1.26–2.28
Family substance use (no)	1.36	0.26	0.99–1.85
Access to care			
Receipt of TANF (no)	0.57 ***	0.12	0.40–0.81
Receipt of SNAP (no)	1.54 **	0.24	1.17–2.03
Receipt of WIC (no)	1.42 *	0.28	1.02–1.97
Receipt of Lunch Program (no)	1.94 ***	0.30	1.50–2.51
Wald’s χ^2^ =	500.77 **		

Notes: *** *p* < 0.01; ** *p* < 0.05; * *p* < 0.1; OR—odds-ratios; RSE—robust standard errors; CI—Confidence-interval; reference groups are in parentheses.

## Data Availability

The original contributions presented in this study are included in the article. Further inquiries can be directed to the corresponding author.
